# Imputation methods for missing data for polygenic models

**DOI:** 10.1186/1471-2156-4-S1-S42

**Published:** 2003-12-31

**Authors:** Brooke Fridley, Kari Rabe, Mariza de Andrade

**Affiliations:** 1 Department of Statistics, Iowa State University, Ames, Iowa, USA; 2 Division of Biostatistics, Department of Health Sciences Research, Mayo Clinic, Rochester, Minnesota, USA; 3 Department of Mathematics, University of Wisconsin-La Crosse, La Crosse, Wisconsin, USA

## Abstract

Methods to handle missing data have been an area of statistical research for many years. Little has been done within the context of pedigree analysis. In this paper we present two methods for imputing missing data for polygenic models using family data. The imputation schemes take into account familial relationships and use the observed familial information for the imputation. A traditional multiple imputation approach and multiple imputation or data augmentation approach within a Gibbs sampler for the handling of missing data for a polygenic model are presented.

We used both the Genetic Analysis Workshop 13 simulated missing phenotype and the complete phenotype data sets as the means to illustrate the two methods. We looked at the phenotypic trait systolic blood pressure and the covariate gender at time point 11 (1970) for Cohort 1 and time point 1 (1971) for Cohort 2. Comparing the results for three replicates of complete and missing data incorporating multiple imputation, we find that multiple imputation via a Gibbs sampler produces more accurate results. Thus, we recommend the Gibbs sampler for imputation purposes because of the ease with which it can be extended to more complicated models, the consistency of the results, and the accountability of the variation due to imputation.

## Background

Methods to handle missing data have been a statistical area of research for many years [1]. Little has been done within the context of pedigree analysis. The goals of this paper are: 1) to present two imputation methods for missing phenotype information, and 2) to compare estimates of the additive polygenic effect using variance components or mixed models between the Genetic Analysis Workshop 13 (GAW13) simulated missing phenotype and the complete phenotype data sets for each imputation method [2-4]. In narrowing the focus of our topic, we only looked at the phenotypic trait systolic blood pressure and the covariate gender at time point 11 (1970) for Cohort 1 and time point 1 (1971) for Cohort 2. The methods for imputation described herein include traditional multiple imputation and multiple imputation (data augmentation) via a Gibbs sampler, with both methods accounting for the familial information in the imputation. In fitting the polygenic model to produce estimates of the overall mean effect, gender effect, additive genetic variance, and the residual error variance, we used the expectation-maximization (EM) algorithm program PolyEM [5] and a Bayesian analysis involving use of a Gibbs sampler.

## Methods

### Traditional multiple imputation method

Multiple imputation is carried out by using the conditional distribution of the missing values given the observed values. Let **Y**_i _= (**Y**^T^_mis_, **Y**^T^_obs_)^T ^be the quantitative phenotype values for the i^th ^family with **Y**_mis _representing the vector of missing values and **Y**_obs _representing the vector of observed values. Similarly, we can partition the mean and covariance matrix. Thus, for the polygenic model (including an overall mean and a gender effect), the distribution of **Y**_mis _given **Y**_obs _is

**Y**_mis _| **Y**_obs _~ N(**μ**_1_, Σ_1_),

with **μ**_1 _= (**μ **+ X_1_**β**) + (σ^2 ^D_12_)(σ^2 ^D_22 _+ τ^2 ^I_22_)^-1^(**Y**_obs _- (**μ **+ X_2 _**β**))

and Σ_1 _= (σ^2 ^D_11 _+ τ^2 ^I_11_) - (σ^2 ^D_12_)(σ^2^D_22 _+ τ^2 ^I_22_)^-1 ^(σ^2 ^D_21_).

The imputation is carried out by generating missing values from the conditional multivariate normal distribution, taking into account the family structure. This imputation is completed *m *times to produce *m *complete data sets to analyze. From these *m *analyses, the final point estimate would be the mean of the *m *estimates. The computation of the standard error can be done by first computing the between-imputation variation, B_*m*_, and the within-imputation variation, W_*m*_. Then, the total variability is T_*m *_= W_*m *_+ B_*m *_(m + 1)/m. Confidence intervals for parameters of interest use the *t*-distribution with degrees of freedom (m - 1) (1 + 1/ (m + 1)*W_*m*_/B_*m*_)^2 ^[6].

The one problem with this imputation scheme is that values for μ, **β**, σ^2^, and τ^2 ^are required for the imputation. To address this issue, we ran the analysis on the observed data and used the resulting estimates to create *k *complete data sets, from which *k *sets of point estimates are found. Then, the average of the *k *sets of estimates is used for the *m *multiple imputations. Point estimates were found using the EM algorithm program PolyEM with *k *= 5 and *m *= 25 [5,7]. Variance estimates were found using the Fisher Information matrix and the large sample properties of maximum likelihood estimates (MLEs) [4,7,8].

### Multiple imputation via Gibbs sampler

Another approach for the imputation of missing data is through a Bayesian analysis via a Gibbs sampler. The Gibbs sampler is a particular Markov chain algorithm that is useful when working with high dimensional problems. In addition to the traditional use of the Gibbs sampler, an imputation step can also be added to impute missing values. A multiple imputation scheme can be implemented by having an imputation step at the beginning of the Gibbs sampler. For each iteration, a new imputation is done, giving multiple imputations for each missing value [9-15]. For a detailed proof of the data augmentation methodology, see Tanner and Wong [12].

A Bayesian polygenic model with non-informative prior distributions for the i^th ^family is **Y**_i _= X_i_**β **+ **a**_i _+ **ε**_i_, where **Y**_i _is a vector containing the individual responses in family i, X_i _is a design matrix containing covariate information, **β **is a vector of covariate effects, **a**_i _is a vector of random family effects where **a**_i _~ MVN(0, σ^2^D_i_) and D_i _is a known coefficient of relationship matrix, and **ε**_i _~ MVN(**0**, τ^2^I). Non-informative priors were then placed on all other parameters in the model, i.e., p(**β**) proportional to **1**, p(σ^2^) proportional to 1/σ^2^, and p(τ^2^) proportional to 1/τ^2 ^[6].

The steps for implementing the Gibbs sampler with an imputation step for the Bayesian polygenic model follow.

1. Set starting values for **β**^(0)^, σ^2(0)^, τ^2(0)^, **a**_i_^(0) ^for all i = 1,..., k, and set m = 1 (iteration).

2. If y_ij _is missing, impute y_ij _by simulating an observation from N(**X**_ij _**β **^(m-1) ^+ a_ij_^(m-1)^, τ^2(m-1)^).

3. Generate **β**^(m) ^from MVN((X^T^X)^-1^X^T^(**y **- **a**^(m-1)^), τ^2(m-1)^(X^T^X)^-1^).

4. Generate τ^2(m) ^from INGAM(N/2, 1/2 Σ(**y**_i_-X_i_**β**^(m) ^- **a**_i_^(m-1)^)^T^(**y**_i_-X_i_**β**^(m) ^- **a**_i_^(m-1)^)).

5. Generate σ^2(m) ^from INGAM(N/2, 1/2 Σ **a**_i_^(m-1)T ^D_i_^-1^**a**_i_^(m-1)^).

6. Generate **a**_i_^(m) ^from MVN(**μ**_a_^(m)^, V_a_^(m)^), where **μ**_a_^(m) ^= (1/σ^2(m) ^* D_i_^-1 ^+ 1/τ^2(m)^* I_i_)^-1 ^* (1/ τ^2(m) ^* I_i _(**y**_i _- X_i_**β**^(m)^)) and V_a_^(m) ^= (1/σ^2(m) ^* D_i_^-1 ^+ 1/τ^2(m) ^* I_i_)^-1^.

7. After one iteration of the algorithm, you have (**β**^(m)^, σ^2(m)^, τ^2(m)^, **a**^(m)^). Set m = m + 1 and repeat steps 1 through 6.

Approximate (1 - α)% posterior confidence intervals are found by taking the α/2 and the 1-α/2 percentiles of the simulated marginal posterior distributions for parameters of interest. The simulated posterior distributions will reflect the uncertainty in the estimation of the parameter along with the uncertainty due to the imputation of the missing data.

## Results

The two imputation methods were run for three replicates of the GAW13 simulated complete and missing data sets for the phenotypic trait of systolic blood pressure and the covariate of gender at time point 11 (1970) for Cohort 1 and time point 1 (1971) for Cohort 2. We limited our analysis to two odd-numbered replicates and one even-numbered replicate to demonstrate the methods due to time and computational restrictions. Table 1 displays the results for the GAW13 missing and complete data sets, in which a likelihood analysis using an EM algorithm using the traditional imputation approach for the missing data. Table 2 displays the results for the GAW13 missing and complete data sets, in which a Bayesian model was used involving a data augmentation step for missing data.

The confidence intervals show little difference between the complete and missing data set analysis within each type of imputation method with regards to the estimates for the error variance component. The data augmentation within a Gibbs sampler gives intervals similar to those of the complete data set, while the traditional imputation approach for missing data produced differing intervals when compared with the complete data set analysis. Not only are the confidence intervals narrower after the imputation of missing data, but the point estimates for the polygenic variance component are much smaller (underestimated) as compared to the results of the complete data sets for the traditional imputation method. These intervals show that the traditional imputation method produced inaccurate intervals as compared with the intervals for the complete data set. The inaccuracy may be due to the choice of the parameter values for the imputation or the degrees of freedom used for the intervals [16].

## Conclusions

Missing data complicates statistical analysis. One way to deal with missing data is through the use of imputation. In the case of family data, the information provided by the dependence structure can be utilized in the imputation of missing data. We have discussed two methods of imputation and illustrated their application through the GAW13 simulated data sets. We assumed the missing data mechanism in the GAW13 data set was ignorable. The Gibbs sampler and the traditional multiple imputation method can be easily applied to non-ignorable missing data, such as in cases involving censored phenotype data. In the case of non-ignorable missingness, not adjusting for the missing/censored values will lead to biased estimates. Based on results from the three replicates, the Gibbs sampler approach seems to give more accurate confidence intervals, as opposed to the traditional multiple imputation approach. In addition, the traditional imputation method has the drawback of needing parameter values for the completion of the imputation. The Gibbs sampler approach does not encounter this problem, because any set of estimates may be used as starting values for the algorithm. In conclusion, we recommend the Gibbs sampler for imputation purposes because of the ease with which it can be extended to more complicated models, the consistency of the results, and the accountability of the variation due to imputation. Future work is planned to investigate the use of data augmentation for missing phenotype data in longitudinal studies and quantitative trait locus analysis.

## Figures and Tables

**Table 1:** 95% Confidence intervals using "traditional" multiple imputation within a likelihood analysis for the complete and missing simulated data

	**Missing Data**		**Complete Data**	
		
**Rep**	**Polygenic VC**	**Error VC**	**Polygenic VC**	**Error VC**
003	(77.68, 90.99)	(152.25, 189.97)	(86.08, 133.07)	(145.59, 188.12)
004	(71.29, 96.34)	(151.42, 190.75)	(81.58, 127.39)	(151.10, 189.15)
019	(75.86, 88.50)	(154.40, 188.91)	(65.23, 107.04)	(154. 90, 191.57)

**Table 2:** 95% Approximate posterior intervals using data augmentation within a Bayesian/Model for the complete and missing simulated data

	**Missing Data**		**Complete Data**	
		
**Rep**	**Polygenic VC**	**Error VC**	**Polygenic VC**	**Error VC**
003	(90.38, 129.60)	(148.30, 182.30)	(88.47, 131.90)	(151.50, 186.30)
004	(80.30, 122.50)	(147.90, 183.20)	(86.75, 126.70)	(153.40, 185.40)
019	(67.49, 95.44)	(157.50, 188.10)	(71.13, 104.80)	(158.90, 189.30)
